# An update on the clinical diagnostic value of β-hCG and αFP for intracranial germ cell tumors

**DOI:** 10.1186/s40001-016-0204-2

**Published:** 2016-03-12

**Authors:** Mingming Hu, Hongzhi Guan, Ching C. Lau, Keita Terashima, Zimeng Jin, Liying Cui, Yuzhou Wang, Guilin Li, Yong Yao, Yi Guo, Yan Michael Li, Dingrong Zhong, Juan Xiao, Xirun Wan, Xin Lian, Feng Feng, Haitao Ren, Yanhuan Zhao, Xinqi Cheng, Feng Gu

**Affiliations:** Department of Endocrinology, Peking Union Medical College Hospital, Chinese Academy of Medical Science, Key Lab of Ministry of Health, Beijing, 100730 China; Department of Neurology, Peking Union Medical College Hospital, Chinese Academy of Medical Science, Beijing, 100730 China; Texas Children’s Cancer and Hematology Centers, Baylor College of Medicine, Houston, TX 77030 USA; Children’s Cancer Center, National Center for Child Health and Development, Tokyo, 157-8535 Japan; Department of Oncology, Peking Union Medical College Hospital,Chinese Academy of Medical Science, Beijing, 100730 China; Department of Neurosurgery, Beijing Xuanwu Hospital, Beijing, 100053 China; Department of Neurosurgery, Peking Union Medical College Hospital, Chinese Academy of Medical Science, Beijing, 100730 China; Department of Neurosurgery and Oncology, University of Rochester, Rochester, NY 14642 USA; Department of Pathology, Peking Union Medical College Hospital, Chinese Academy of Medical Science, Beijing, 100730 China; Department of Pediatrics, Peking Union Medical College Hospital, Chinese Academy of Medical Science, Beijing, 100730 China; Department of Gynaecology and Obstetrics, Peking Union Medical College Hospital, Chinese Academy of Medical Science, Beijing, 100730 China; Department of Radiotherapy, Peking Union Medical College Hospital, Chinese Academy of Medical Science, Beijing, 100730 China; Department of Radiology, Peking Union Medical College Hospital, Chinese Academy of Medical Science, Beijing, 100730 China; Department of Clinical Laboratory, Peking Union Medical College Hospital, Chinese Academy of Medical Science, Beijing, 100730 China

**Keywords:** Intracranial germ cell tumors, Tumor markers, Chorionic gonadotropin, Beta subunit, Human, Alpha-fetoproteins

## Abstract

**Background:**

Pathological examination combined with tumor markers has become a standard for the diagnosis of intracranial germ cell tumors (ICGCTs), but the current concept of ‘secreting germ cell tumors’ and three empirically highly specific diagnostic criteria (β-hCG ≥ 50 IU/L or αFP ≥ 10 ng/mL; β-hCG ≥ 100 IU/L or αFP ≥ 50 ng/mL; β-hCG > 50 IU/L or αFP > 25 ng/mL) are not based upon pathology examination or CSF cytology. Further investigation is needed to re-evaluate their value.

**Methods:**

A multidisciplinary diagnostic team was created. Valid β-hCG/αFP data were collected from cases of ICGCTs confirmed by pathology and CSF cytology (*n* = 58) between 1991 and 2012, and from suspected ICGCTs cases (*n* = 17) between 2011 and 2012 as controls [Langerhans cell histiocytosis (LCH), *n* = 12; and other intracranial tumor (ICT), *n* = 5]. The cut-off points for β-hCG and αFP were calculated using receiver operating characteristic (ROC) curves.

**Results:**

This study clarifies the relative rationality of one criteria (β-hCG > 50 IU/L and αFP > 25 ng/mL); confirms new β-hCG diagnostic cut-off points: CSF β-hCG ≥ 8.2 IU/L and serum β-hCG ≥ 2.5 IU/L (sensitivity of 47 and 34 %, respectively, specificity of 100 %, both; *P* < 0.05); and empirically adjusts the criteria for αFP to ≥ 3.8 ng/mL in CSF and to ≥ 25 ng/mL in serum. The total diagnostic sensitivity for ICGCTs finally increased from 34.6 to 65.4 % (*P* < 0.05, diagnostic value of CSF β-hCG exceeds 90 %). Subtype diagnosis improved with αFP in 16.7 % of non-geminomatous germ cell tumor cases.

**Conclusion:**

New evidence-based criteria of β-hCG and αFP can help improving early and formal diagnosis of ICGCTs, and is of great clinical significance.

## Background

Whatever their origin, intracranial germ cell tumors (ICGCTs) are classified into germinomas (including simple germinomas and syncytial trophoblast giant cells (STGCs); >82 % of ICGCTs) and non-geminomatous GCTs (NGGCTs) (including embryonal carcinomas, yolk sac tumors, choriocarcinomas, teratomas, and mixed germ cell tumors) [1]. About 40–46 % of ICGCTs are in the pineal region, while 30–42 % are in the sellar region [2]. ICGCTs are more common in males than females (ratio of about 4 to 1) and generally occur in people <30 years old with a peak incidence at 10–12 years [3]. Although ICGCTs are quite rare, it was previously thought that ICGCTs were more common in Asia than in Europe or the USA [1].

Historically, it was found that certain ICGCTs are capable of secreting tumor markers such as the β subunit of human chorionic gonadotropin (β-hCG) and/or α-fetoprotein (αFP). As a result, a 1990s consensus defined NGGCTs as “secreting germ cell tumors” [4, 5], but this concept has been found to be limited. First, many pathologically confirmed cases of germinoma are showing highly variable levels of β-hCG in the cerebrospinal fluid (CSF) and serum [6–9]. Secondly, no criteria have been agreed upon these levels, but three criteria are used for diagnosis and initiating chemotherapy, all three using the same levels in either the serum or CSF. These criteria evolved since the 1990s and are (1) β-hCG ≥ 50 IU/L or αFP ≥ 10 ng/mL [4, 5, 10–12]; (2) β-hCG ≥ 100 IU/L or αFP ≥ 50 ng/mL [13]; and (3) β-hCG > 50 IU/L or αFP > 25 ng/mL [14]. Finally, these criteria fail to present the meaning of differential diagnoses among other suspected intracranial lesions located in classical sites of ICGCTs (such as the pineal region, the sellar region, and the basal ganglia region). More appropriate diagnostic criteria should also help diagnosing the ICGCTs at an early stage to avoid a delayed diagnosis [15–18].

Therefore, it is of great clinical significance to re-evaluate the clinical diagnostic criteria of β-hCG and αFP levels. The aim of this study was to investigate the levels of β-hCG and αFP in CSF and serum with pathological examination (as the gold standard) in order to improve the early and standardized diagnosis of different subtypes of ICGCTs, not only the classical “secreting germ cell tumors.”

## Methods

### Study subjects

This study was approved by the Ethics Committee of the Peking Union Medical College Hospital (PUMCH). As detection of these tumor markers is included in the routine clinical laboratory work-up of patients suspected with ICGCT, consent to treatment was signed by the patients or their guardians, but the committee waived the need for individual research consent.

The ICGCT study population included only the cases of ICGCT confirmed by histopathology and/or CSF cytology and admitted at the PUMCH between March 1991 and December 2012. Patients were excluded if they had already undergone radiation therapy before surgery, had been diagnosed only by tumor markers, or had an associated intracranial infection.

In the ICGCT group, 26 cases were prospectively collected between September 2011 and December 2012, which was defined as the “recent” group, while the cases collected between 1991 and 2011 were defined as the “past” group. All patients in the recent group had synchronous β-hCG and αFP data, while only part of the past group had synchronous available data.

A positive control population was selected from cases with pathologically confirmed Langerhans cell histiocytosis sellar region lesions (LCH group, *n* = 12), including four patients with saddle area LCH, and intracranial tumors (ICT group, *n* = 5), all admitted at the PUMCH between September 2011 and December 2012. All patients in the control group had synchronous β-hCG and αFP data.

### Study design

**Fig. 1:**
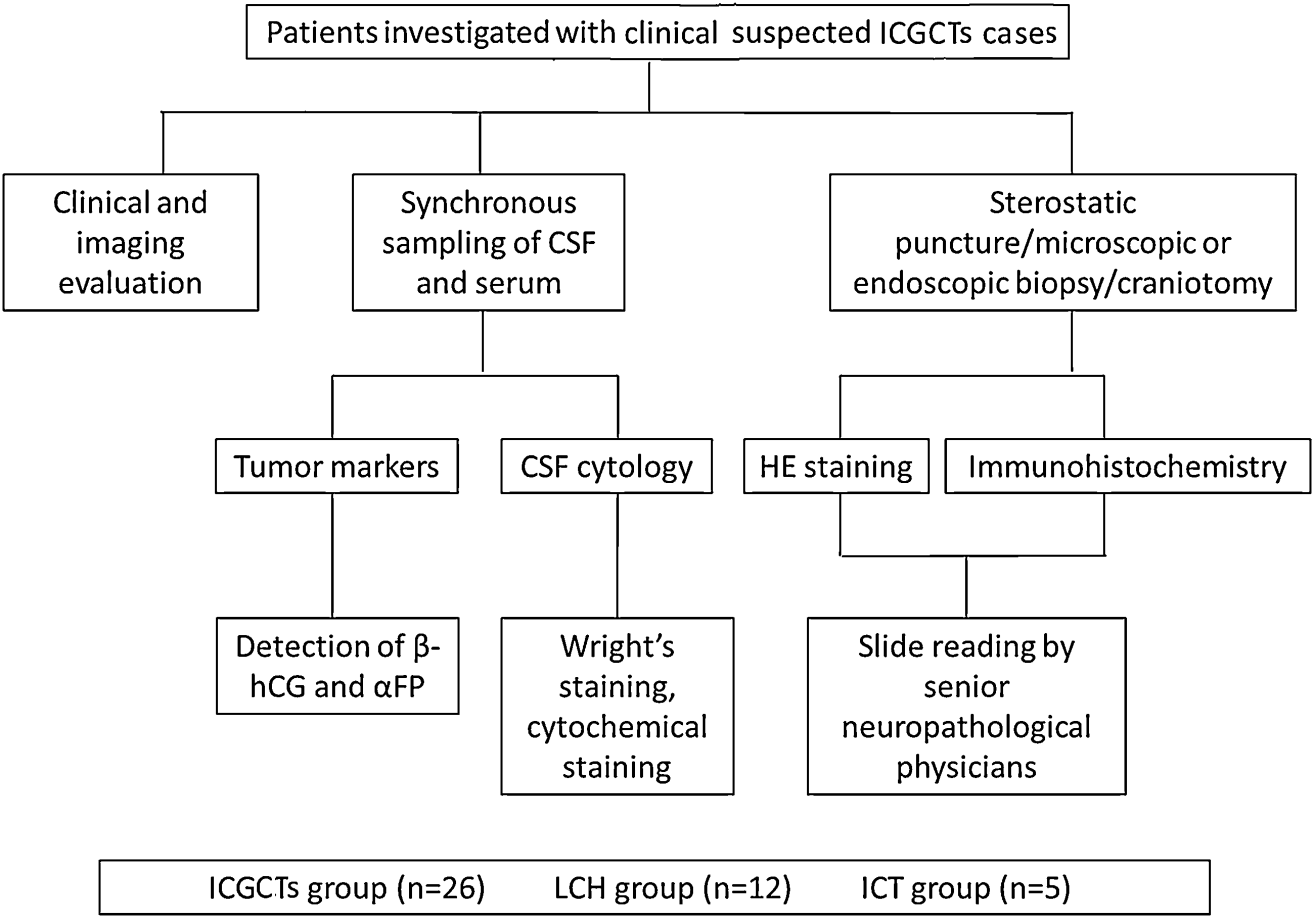
Diagnostic algorithm for intracranial germ cell tumor (ICGCTs). *CSF* cerebrospinal fluid, *HE* hematoxylin and eosin, *β*-*hCG* β-subunit of human chorionic gonadotropin, *αFP* alpha fetoprotein, *LCH* langerhans cell histiocytosis, *ICT* intracranial tumor

A comprehensive multidisciplinary diagnostic team for ICGCTs was created before collecting the patients from the recent period. The diagnostic algorithm is shown in Fig. 1.

The demographic data, tumor site, surgery pattern/surgical access, degree of excision, results of histopathology and CSF cytology, and staging of CSF dissemination were collected. Both the latest β-hCG and αFP data from lumbar puncture CSF and serum were gathered before the cases were confirmed in order to examine, compare, and evaluate three diagnostic criteria of “secreting tumors” through the strategy of exclusion. During this process, the data was available but not necessary “synchronous.”

The term “synchronous” had different meanings between different groups. In the past group, it meant that the interval of obtaining CSF and blood serum samples was within 24 h. In the recent and control groups, it meant that both CSF and blood serum samples were collected at the same time.

“Valid” data were defined for early ICGCT diagnosis by exploring the lower cut-off values compared to the current criteria. Valid β-hCG data meant that it had to be under 50 IU/L for early diagnosis, while valid data for αFP (<10, <25, or <50 ng/mL) was only determined after the proper criteria by examination of the earlier αFP data. Before the cases were confirmed, both the lumbar puncture CSF and serum β-hCG and αFP data were gathered to evaluate the three diagnostic criteria of secreting tumors according to the pathological and CSF cytological results (not all data were synchronous). Measurement detection thresholds for β-hCG and αFP were 21 U/L and 0.605 ng/mL, respectively. Therefore, by adding 2 or 1 to the treatment data to the original value, the value of 0 is avoided and can be used with the natural logarithm. After being added with 2 (for β-hCG) or 1 (for αFP), both groups underwent natural logarithmic transformation to observe the distributions of the two tumor markers in CSF and blood serum as well as the correlation analysis and the linear regression analysis (all data were synchronous). Two valid groups were selected to combine with the β-hCG and αFP data of lumbar puncture CSF and serum in the LCH group and the other intracranial tumors group for ROC analysis. The possible diagnostic cut-off values of β-hCG and αFP were then calculated in both CSF and serum. Both cut-off values were compared with the diagnostic criteria of secreting tumors to assess their diagnostic significance.

### Tumor evaluation criteria

Regional classifications: all lesions that involved one kind of basal ganglia region were classified as basal ganglia lesions, while the others were classified into the pineal lesions and sellar lesions. The mixed type was defined by two or more non-adjacent lesions but without basal ganglia region involvement.

Surgical extent was classified as biopsy (<10 % of the lesion), partial resection (PR; 10–49 % of the lesion), total resection (GTR; microscopic total resection), and subtotal resection (STR, between 50 % and total resection).

Germ cell tumors were classified as NGGCTs and mixed germ cell tumors (diagnosed by two or more confirmed subtypes, or significantly increased αFP concentration in CSF or blood serum though other tumor cells were not clear except for the content of the germinoma).

CSF dissemination staging was classified based on the complete absence of tumor cells in the CSF (M0), the presence of tumor cells in the CSF (M1), implantation metastases with tubercles in the ventricular system or cranial subarachnoid space (M2), implantation metastases with tubercles in the spinal subarachnoid space (M3), and extracranial metastases (M4).

### Evaluation of tumor markers

The methods for detecting tumor markers changed slightly during the study period. The PUMCH kept using the ADVIA Centaur Total HCG ReadyPack ELISA kit (detection threshold of 2.0 IU/L) for β-hCG that was originally provided by Bayer Healthcare Pharmaceuticals (Montville, NJ, USA). This test was then acquired by Siemens (Erlangen, Germany) in 2005, without change in the detection threshold. The same test was used throughout the study period. Initially, αFP was detected by an electrochemiluminescence immunoassay (ECLIA) from Roche Diagnostics (Basel, Switzerland), and the instrument was changed in 2010 to a Cobas 601 (Roche Diagnostics, Basel, Switzerland), which uses the same methodology and has the same detection threshold of 0.605 ng/mL.

### Statistical analysis

SPSS 22 (SPSS Inc., Chicago, IL, USA) was used for data management, statistical analyses, and creating figures. Continuous variables are presented as mean ± standard deviation or median (range), as appropriate. An independent samples *t* test was applied to pairs of normally distributed continuous data. Correlation and linear regression analyses were performed on non-normally distributed continuous data by adding 2 or 1 (β-hCG: +2, αFP: +1) and was transformed by natural logarithm. Categorical data were analyzed using the Chi-square test (Fisher’s exact probability calculation). ROC curve analysis was applied to determine the diagnostic cut-off values. Two-sided *P* values <0.05 were considered significant.

## Results

### Characteristics of the patients

There were 26 patients in the recent group and 32 in the past group. Significant differences were found between the two groups for surgical procedures, tumor sites, and pathological classifications. Therefore, the two populations were divided into two different groups in terms of background characteristics (Table 1). No specific sequelae or severe disabilities were observed in the two groups.

**Table 1:** Characteristics of the recent and past groups of patients with intracranial germ cell tumors

Items		Past group (*n* = 32)	Recent group (*n* = 26)		
Patients demographics					
Sex ratio (male:female)		17:15 (1.1:1)	17:9 (1.9:1)		
Age at diagnosis (years), mean ± SD (range)^*^		19 ± 6.9 (9–41)	12.7 ± 4.3 (6–24)		
Tumor characteristics					
Pathological classification, *n* (%)	Germinoma	31 (90.6)	17 (65.4)		
	NGGCTs	0 (0)	2 (7.7)		
	Mixed type	1 (9.4)	7 (26.9)		
Tumor location, *n* (%)^a^	Pineal	1 (3.1)	4 (15.3)		
	Sellar	19 (59.4)	10 (38.5)		
	Basal Ganglia	4 (12.5)	6 (23.1)		
	Mixed type	8 (25)	6 (23.1)		
M staging (cases)	M0 (22/16)	M1 (4/1)	M2 (4/9)	M3 (2/0)	M4 (0/0)
Method of confirmation					
CSF cytology, *n* (%)	Total cases	14 (43.8)	26 (100)		
	Positive	9 (64.3)	1 (3.8)		

Surgical resection		Germinoma	NGGCTs	Mixed type	Total
	Biopsy	8/16	0/0	0/2	8/18
	PR	7/0	0/0	1/1	8/1
	STR	7/1	0/2	0/1	7/4
	GTR	0/0	0/0	0/2	0/2
	No surgery	9/1	0/0	0/0	9/1
History of radiography (cases)		0/1	0/1	0/1	0/3

*NGGCTs* non-geminomatous germ cell tumors, *CSF* cerebrospinal fluid; *PR* partial resection, *STR* subtotal resection, *GTR* total resection, *M* staging: metastasis staging)

^*^ *P* < 0.05 recent vs. the past groups

^a^Both groups showed that the sellar region was the most common site, with a higher proportion in females; in addition to the classic bifocal tumors (sellar and pineal), there were other types of bifocal tumors (basal ganglia and sellar region) as well as triple lesions (pineal, sellar, and basal ganglia)

Only one case in the past group was a mixed tumor (contained germinoma and mature teratoma). The recent group comprised all ICGCT subtypes including embryonal carcinoma, yolk sac tumor, choriocarcinoma, and all types of teratoma (mature, immature, and malignant transformation) as well as one rare and never reported case of pineal intermediate trophoblastic tumor (ITT). In addition, one case of mixed pineal tumor was associated with sellar region pituicytoma. There was no patient staged as M4, while only the past group had M3 patients that involved the cervical spinal cord as well as cervical, lumbar, and sacral nerve roots.

Results of CSF cytology showed germ cell tumors in both groups, but the positivity rate was higher in the past group than in the recent group; the only case of double lesions in the recent group did not show significant imaging changes: the pituitary stalk was slightly enlarged and the pineal was moderately enhanced. No cytologically positive cases by CSF in either group were given after surgery. Patients in the recent group were all routinely screened by pineal, hypothalamic, and pituitary stalk craniotomies as well as neuroendoscopic pituitary biopsy. Stereotactic puncture was mainly applied to the basal ganglia lesions.

Among controls, there were 12 cases in the LCH group (including four cases of solitary sellar lesion), among which ten patients were male. Mean age at diagnosis was 23.1 years (range 9–51). In the ICT group, five patients were included (three were male) with a median age at diagnosis of 39 (12–57) years. They were two cases of craniopharyngioma, one case of sellar region ganglioglioma, one case of basal ganglia and pineal region primary neuroectodermal tumor, and one case of sellar region metastasis of a lung adenocarcinoma.

### Tumor markers

In the past group, tumor markers were below the detection thresholds in 11 cases (34.4 %), markers were detected in the serum only in five cases (15.7 %), markers were detected in CSF only in one case (3.1 %), and markers were detected in both the serum and CSF in 15 cases (47 %) (including 14 cases of synchronous detection of β-hCG and 13 cases of synchronous detection of αFP). The mean values (range) of CSF and serum β-hCG were 113.5 (1.3–1087.5) IU/L and 16.6 (0–224.5) IU/L, respectively, while the numbers of cases with CSF and serum β-hCG <50 IU/L were 19 and 17, respectively. Mean values (range) of CSF and serum αFP were 4.7 (0.61–34.3) ng/mL and 3.0 (0.71–15.5) ng/mL, respectively.

In the recent group, 26 cases had synchronous detection of β-hCG and αFP in the CSF and serum with mean values (range) of CSF and serum β-hCG 171.1 (4–3060) IU/L and 214.1 (0–4493) IU/L, respectively. There were 13 and 21 cases with CSF and serum β-hCG <50 IU/L, respectively. The mean values (range) of CSF and serum αFP were 7.7 (0.2–147) ng/mL and 53 (0.8–1013) ng/mL, respectively.

In the LCH group, the mean values (range) of CSF and serum β-hCG were 4.3 IU/L (2–6) and 0, respectively, while CSF and serum αFP were 0.55 ng/mL (0.2–0.7) and 2.5 ng/mL (1.0–5.4), respectively.

In the ICT group, the mean value (range) of β-hCG in the CSF was 4.4 (3–8) IU/L, while was 0 in the serum. The mean value (range) of CSF αFP was 0.68 (0.61–0.8) ng/mL, and was 2.7 (1.3–6.1) ng/mL in the serum.

Since the mean values of αFP did not differ greatly between the two groups, we combined all values of αFP to calculate the mean value (range), which was 0.59 (0.2–0.8) ng/mL in the CSF and 2.55 (1–6.1) ng/mL in the serum.

### Evaluation of the current diagnostic criteria

Since the three diagnostic criteria of secreting tumors are all empirical, no sensitivity or specific data were available. However, in this study, we aimed to ensure a diagnostic specificity of 100 % in order to lower the misdiagnosis rate to the utmost extent. For the entire diagnosis procedure, we adopted the “exclusion method.”

For example, in five cases eventually diagnosed with secreting tumors, three (cases 1–3) had CSF β-hCG levels <100 IU/L and four (cases 1–4) had β-hCG serum levels <100 IU/L; hence, diagnostic β-hCG levels >50 IU/L was more rational. However, two cases (cases 1 and 2) had CSF and serum β-hCG levels <50 IU/L; so even β-hCG levels >50 IU/L were not able to replace the pathological examination, but might help avoiding misdiagnosis (Table 2).

**Table 2:** Empirical examination on the diagnostic criteria for secreting tumors

Case number	CSF β-hCG (IU/L)	Serum β-hCG	CSF αFP (ng/mL)	Serum αFP	CSF cytology	Histopathology	Final diagnosis
1	20	3	0.7	15.5	NEG	GERM	GERM
2	20	4.5	2.4	11.4	NEG	GERM	GERM
3	61	12.1	3.8	33.5	NEG	GERM	MIXED
4	252	28.9	8.6	33.5	NEG	EMB	EMB
5	204.6	204.6	19.1	35.6	NEG	MIXED	MIXED

*CSF* cerebrospinal fluid, *β*-*hCG* β-subunit of human chorionic gonadotropin, *αFP* alpha fetoprotein, *NEG* negative, *GERM* germinoma, *MIXED* mixed germ cell tumors, *EMB* embryonal carcinoma

The lowest criterion for αFP diagnosis as secreting tumors was 10 ng/mL. Therefore, two of these cases (cases 1 and 2) were initially considered as NGGCTs (secreting tumors), but the final pathological diagnosis proved them to be germinomas. Moreover, since the three remaining cases (cases 3–5) had CSF/serum αFP levels of 25–50 ng/mL and were diagnosed as NGGCTs, the αFP threshold of >25 ng/mL was relatively rational.

### Correlation between CSF levels and serum tumor markers levels in synchronized samples

**Fig. 2:**
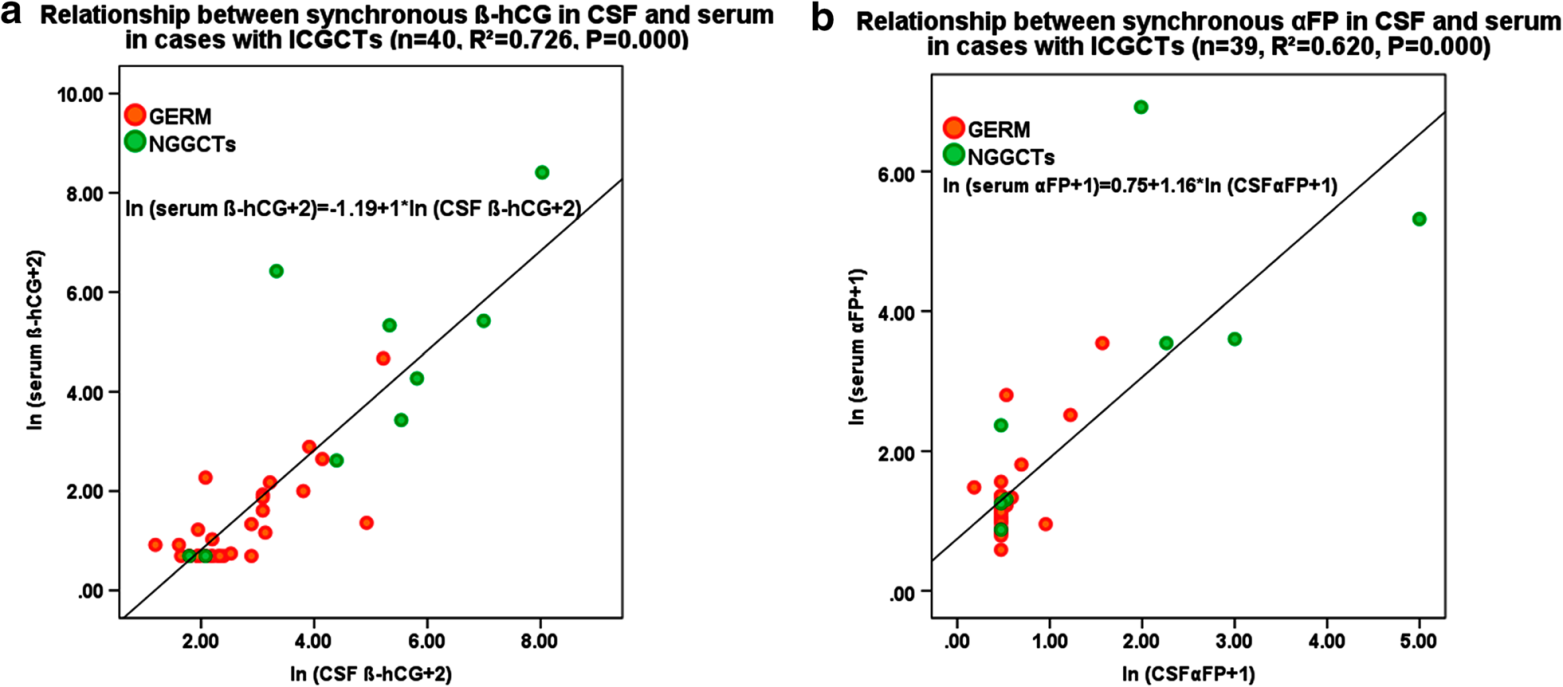
*Scatter diagrams* representing the relationship between synchronously sampled tumor markers in the cerebrospinal fluid (CSF) and serum. The tumors are classified as germinoma (*GERM, red marks*) and NGGCTs (*green marks *). **a** Relationship between β-subunit of human chorionic gonadotropin (β-hCG) levels (*n* = 40, *r* = 0.852, *P* < 0.05). **b** Relationship between alpha fetoprotein (αFP) levels (*n* = 39, r = 0.788, *P* < 0.05)

A total of 40 pairs (14 pairs from the past group and 26 pairs from the recent group) of synchronous data of CSF and serum β-hCG were used to plot a scatter diagram (Fig. 2a), which was linear after logarithmic transformation and was statistically significant (*r* = 0.852, *P* < 0.05). β-hCG levels were higher in the CSF than in the serum. When compared to the NGGCTs subgroups (including the mixed tumors), the germinoma subgroup showed a more significant linear trend.

Figure 2b shows 39 pairs of synchronous data of CSF and serum αFP with a linear correlation αFP levels in the CSF being significantly lower than in the serum. The difference between the germinoma subgroup and NGGCTs was not as significant as compared to that of Fig. 2a.

### Calculation of the cut-off values of different tumor markers in CSF and serum by ROC curve

**Fig. 3:**
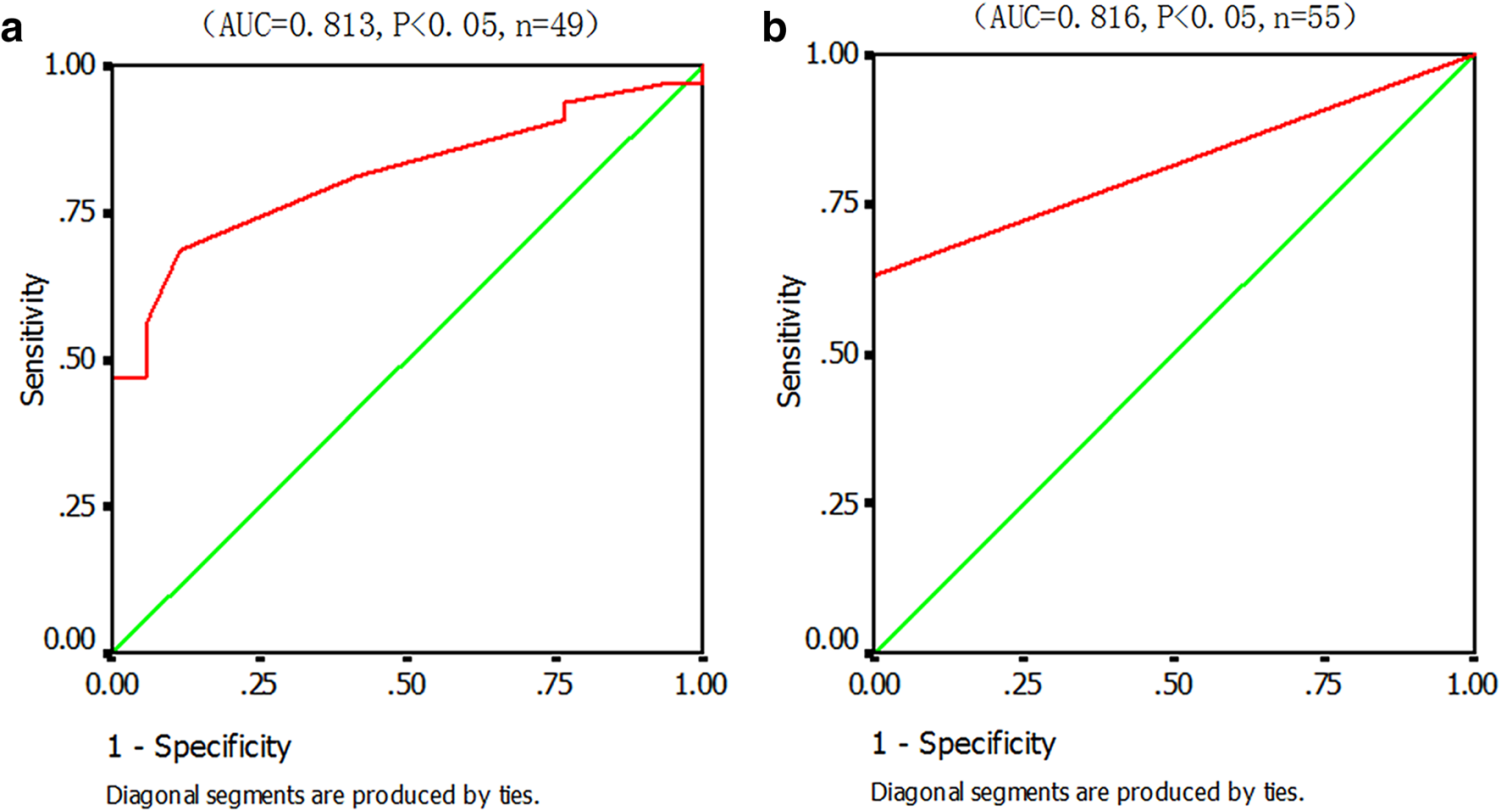
Receiver operating characteristic (ROC) curve analysis of cut-off values for diagnosing intracranial germ cell tumors (ICGCTs). **a** Cerebrospinal fluid (CSF) β-subunit of human chorionic gonadotropin (β-hCG). **b** Serum β-hCG

From Figs. 2 and 3, it was apparent that both CSF and serum should not have the same cut-off values. When calculating the diagnostic cut-off value of CSF β-hCG, priority was given to distinguish significant clinical differences between ICGCTs and LCH, craniopharyngioma, and other tumors.

Figure 3a shows that the use of β-hCG levels in CSF to diagnose ICGCTs was of statistical significance (*P* < 0.05). β-hCG cut-off points had a 100 % specificity at 8.15, 8.65, and 9.75 IU/L. We finally selected ≥8.2 IU/L (the most close value to 8.15) as the diagnostic cut-off value, and sensitivity and specificity were 47 and 100 %, respectively.

The calculation of the diagnostic cut-off value of serum β-hCG included data from 55 cases (Fig. 3b). Figure 3b shows a statistical significance of serum β-hCG levels for diagnosing ICGCTs (*P* < 0.05), but the cut-off value was not the same as in the CSF. Since the serum β-hCG levels in the non-ICGCTs group were all 0 (taking into account the detection sensitivity of 2.0 IU/L), the lowest highly specific diagnostic cut-off value of ≥2.5 IU/L (serum β-hCG cut-off points had a 100 % specificity at 1.85, 2.45 IU/L, and 2.5 was the most close value to 2.45 while above sensitivity—2.0 IU/L) was the only choice to obtain a 100 % diagnostic specificity, resulting in a sensitivity of 34 %.

**Fig. 4:**
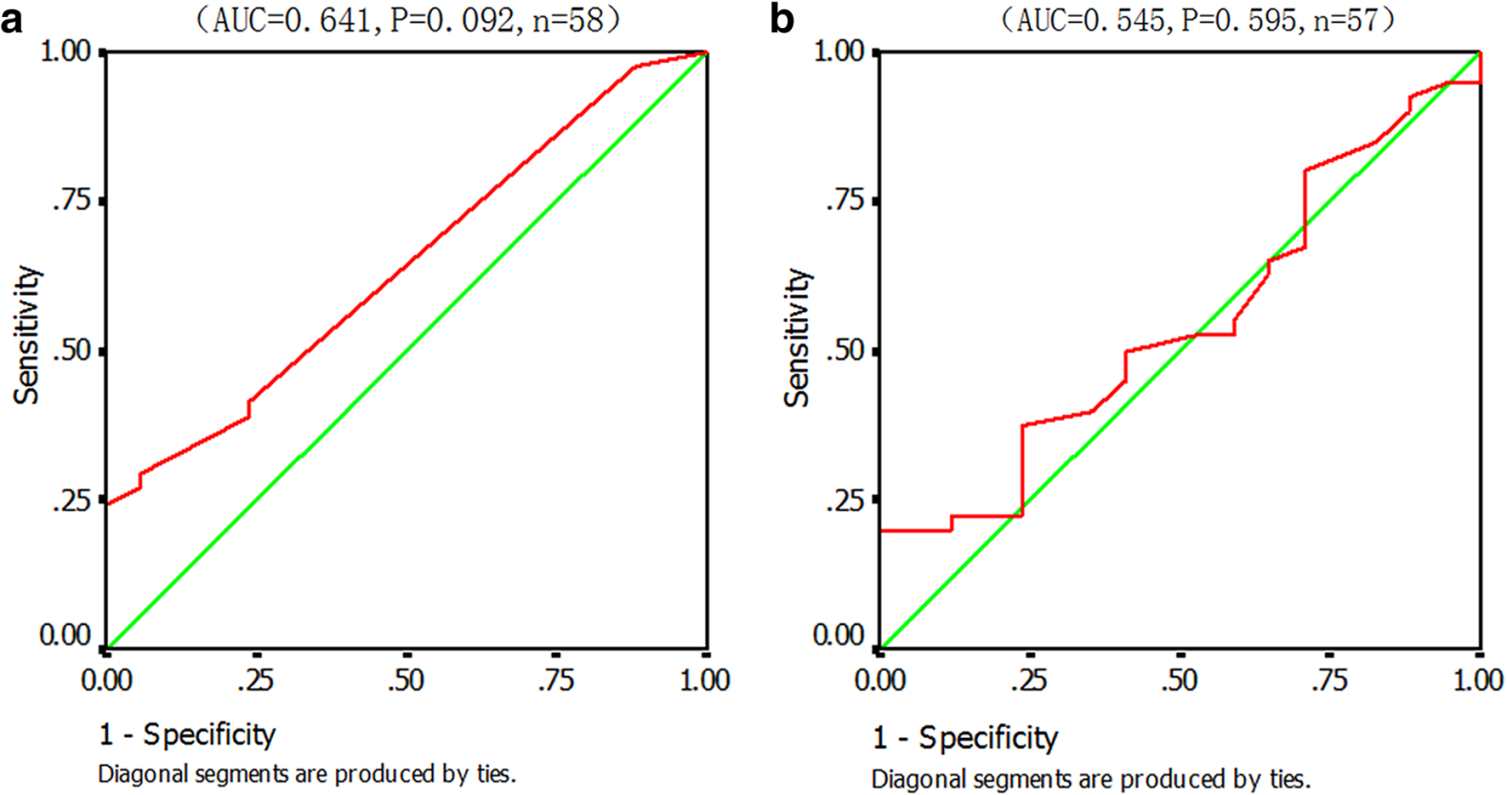
Receiver operating characteristic (ROC) curve analysis of cut-off values for diagnosing intracranial germ cell tumors (ICGCTs) **a** by cerebrospinal fluid (CSF) alpha fetoprotein (αFP) levels and **b** by serum αFP

The calculation for αFP was slightly different from that used for β-hCG because the cut-off value of αFP was considered reasonable at 25 µg/dL, but also because the ROC curve was plotted based on all 57 valid cases. Although no statistically significant diagnostic cut-off values had been found from Fig. 4a, the lowest presumed corresponding cut-off value of CSF αFP levels with 100 % diagnostic specificity was 1.1 ng/mL.

The ROC curve was inapplicable because there was an inadequate amount of valid data of αFP to calculate the diagnostic cut-off values in CSF or serum, but it was possible to perform further empirical deduction. If the serum diagnostic levels of αFP remained at ≥25 ng/mL, the CSF αFP could be downgraded. Based on αFP levels of the three cases (they were 3.8, 6.3, and 8.6 ng/mL, respectively) of the recent group, the diagnostic criterion of CSF αFP was finally identified as being ≥3.8 ng/mL by combining the calculated mean value of αFP of 0.59 ng/mL in the control group as well as the detection sensitivity of αFP of 0.605 ng/mL.

Table 3 shows the new clinical diagnostic criteria of CSF and serum β-hCG and αFP, in comparison with the currently accepted diagnostic criteria (β-hCG > 50 IU/L, αFP > 25 ng/mL). Table 3 also shows that after using the new criteria, the diagnostic sensitivity of β-hCG in both CSF and serum was significantly increased and the CSF β-hCG sensitivity was improved the most. The diagnostic sensitivity of αFP in the CSF increased without any statistical significance.

**Table 3:** Comparison of diagnostic sensitivity between β-hCG and αFP of CSF and blood serum in ICGCTs

	*n*	Old criteria (%)	New criteria (%)	*P* ^a^
CSF β-hCG	42	23.8	59.5	0.002^*^
Serum β-hCG	44	22.7	43.2	0.034^*^
CSF αFP	41	9.8	17.1	0.259
Serum αFP	40	12.5	12.5	–
Total sensitivity^#^	26	34.6	65.4	0.026^*^

*ICGCTs* intracranial germ cell tumors, *CSF* cerebrospinal fluid, *β*-*hCG* β-subunit of human chorionic gonadotropin, *αFP* alpha fetoprotein

* P < 0.05; ^#^ recent group

^a^Fisher’s exact test

When taking into consideration that both β-hCG and αFP may at the same time show positive levels (overlap factor), the total diagnostic sensitivity of tumor markers for the recent group increased from 34.6 % (9/26 cases) to 65.4 % (17/26 cases), among which the major proportion should be attributed to the CSF β-hCG levels (61.5, 94 % cases involved in recent group). Although the diagnostic sensitivity of αFP was lower than that of β-hCG, in the recent group, there were still 16.7 % (1/6 case) diagnosed as mixed ICGCTs and classified into different subtypes based on the serum αFP levels.

## Discussion

Evaluation of the clinical diagnostic value of β-hCG and αFP for ICGCTs is an interesting and confusing topic for endocrinologists, neurologists, and neurosurgeons, and especially for pediatric oncologists. By establishing a multidisciplinary diagnostic team, this study might have established a comprehensive and practical way to solve the problem.

Although the first case was diagnosed by pathological examination in 1991 at the PUMCH, since then, the suspected cases at the PUMCH were mainly diagnosed by diagnostic radiography. However, since the establishment of a comprehensive diagnostic team, we diagnosed 26 cases of ICGCTs in less than 2 years. These cases were similar to those in other countries in terms of epidemiology with the male-to-female ratio of 1.9:1 and mean age of 12.7 ± 4.3 years. In addition, all subtypes have been observed in this study. The frequency of germinoma was 65.4 % and the frequency of sellar lesions was 38.5 %. Moreover, this study found a rare case of ITT, which had not been reported before [19], rather than just study its molecular biology [20, 21]. Secondly, this comprehensive team helped avoid misdiagnosis or mistreatment caused by relying only on diagnostic radiography (especially in solitary sellar LCH cases). Finally, in this study, the representative sample of ICGCTs also had the typical presentation of β-hCG and αFP levels. Since the highest levels of CSF β-hCG were 6 and 8 IU/L in LCH and craniopharyngioma cases, this indicated that these tumor markers in the control group were of diagnostic value.

Although no researchers have given a reasonable explanation of the slight increase of β-hCG levels in LCH and other cases, it is still possible to conclude that the syncytial trophoblast giant cells (STGC) are not the only source of endogenous β-hCG. In fact, this may be related to the detection methods of β-hCG. Although hCG is a glycoprotein with a molecular weight of about 45 kD and has α and β subunits, the germinoma may also independently secrete the β subunit in the free form. Therefore, the National Academy of Clinical Biochemistry (NABC) emphasizes that the detection of β-hCG should include the whole hCG and free β subunit at the same time [22]. Furthermore, some tumors such as trophoblastic tumors of the ovary, gastrointestinal tract, and head and neck may produce hCG, they express moderate levels of β-hCG, and the detection of β-hCG may suffer from false-positive results. The increase of CSF β-hCG in craniopharyngioma reported by Honegger et al. [23] was caused by the detection of β-hCG rather than whole hCG (detected by immunoradiometric assay).

In this study, we have for the first time shown the relative rationality of β-hCG > 50 IU/L or αFP > 25 ng/mL using an exclusion strategy. Gonzalez-Sanchez [8] and Allen et al. [7, 9] showed that CSF β-hCG levels ≥50 IU/L were too high and might result in missed diagnoses of ICGCTs, highlighting the need to develop more optimal diagnostic criteria.

In 2012, Qaddoumi et al. [21] proved for the first time that both β-hCG and αFP had logarithmic linear correlations in CSF and serum, and the total effect showed that β-hCG levels were higher in the CSF compared to the serum, but that αFP was higher in the serum (without stated detection method or sensitivity). This study drew the same conclusion and further inferred that for these two tumor markers, different diagnostic criteria should be used in the CSF or the serum. In this study, β-hCG and αFP levels were more significant in diagnosing early ICGCTs in the CSF rather than in the serum.

In this study, the obtained 8.2 IU/L threshold was quite close to that obtained by Allen [5, 7] (6.8 IU/L, ranging within 1.6–18.7). Nevertheless, the latter only analyzed 60 cases of germinomas, their β-hCG levels were all lower than 50 IU/L, and they did not plot ROC curves against a control group to improve the diagnostic criteria (neither did they state the detection method).

In the normal population, according to the reports of Gonzalez-Sanchez [8] and Tian et al. [24], the upper limit of the 95 % confidence interval for CSF β-hCG levels was 0.688–0.7 IU/L and the maximum value was 0.8–1.330 IU/L (both detected by Roche ECLIA with a detection threshold of 0.1 IU/L). Therefore, considering the consistency among major detection corporations and the marked difference between the normal value and 8.2 IU/L, the cut-off value obtained in this study was significant enough to distinguish between suspicious and real ICGCTs cases.

The cut-off β-hCG value of 2.5 IU/L obtained from serum in ICGCTs seemed to overlap with the normal range <5–10 IU/L [22], but according to the recommendations of the NABC, the age and sex differences of the patients should be taken into consideration since the upper limit was 5 and 3 U/L for menopausal and menstrual females, respectively, and 0.7 and 2.1 U/L for males aged under and over 50 years, respectively, when using a highly sensitive method [22].

In contrast to the calculation for β-hCG, since only a few ICGCT secreted αFP, this study failed to obtain precise diagnostic criteria, but inference analysis revealed that serum levels of 25 ng/mL and CSF levels of 3.8 ng/mL could be used. Although most liver cancers, 10–30 % of intestinal tumors, and even benign liver lesions (such as hepatitis) cause increase serum αFP, they rarely associate with ICGCTs [25]. According to the study by Qin et al. [26], the normal 95 % upper limit of serum levels for Chinese Han people was only 5.76 ng/mL and was positively correlated with age. Therefore, unless new evidence-based cut-off values are found, 25 ng/mL might be a reasonable threshold. For CSF αFP levels, Shi et al. [27] reported a normal 95 % upper limit of 0.968 ng/mL (detected by Roche ECLA with a detection threshold of 0.605 ng/mL). This value is quite close to the cut-off value of 1.1 ng/mL observed in Fig. 4. Therefore, considering the important differences between 1 and 3.8 ng/mL, the diagnostic level of αFP should be decreased when using 3.8 ng/mL.

The new criteria not only markedly increased the clinical diagnostic rate of ICGCT (the diagnostic sensitivity rose from 34.6 to 65.4 %) and helped early diagnosis (decreasing the diagnostic criteria of β-hCG and αFP should increase the early treatment rate), but it also emphasized the important value (over 90 %) of CSF β-hCG levels in the diagnosis and the complementary role of αFP (improving the subtype diagnosis in 16.7 % NGGCTs cases). This procedure should be given attention in clinical practice.

Because the sensitivity of the clinical diagnostic value of αFP and β-hCG for ICGCTs is still not high enough, new markers have been investigated such as CSF microRNA 371–371 and 302 levels, which were significantly increased in ICGCTs, but decreased significantly after treatment; therefore, this marker might be used for further identification of germinoma and NGGCTs [10]. Although microRNAs show some promising value, the αFP and β-hCG levels are currently irreplaceable.

This study has some limitations. First, only a few controls were included and future studies should also include suspected ICGCTs cases (such as pineal blastoma, pineocytoma, glioma [28] or lymphoma or glioma of the basal ganglia region [29]) or children’s craniopharyngioma of the sellar region [30, 31]. Second, we did not explore the distribution of the αFP and β-hCG levels when the brain-blood barrier is injured. Third, we did not compare the relationship between high β-hCG levels (which may cause a β-hCG-dependent precocious puberty in boys or hypogonadotrophin-hyperandrogenemia in older male cases) and the new diagnostic criteria. Fourth, the number of patients was relatively small and these results should be confirmed using multicenter studies. Finally, we chose to set the specificity to 100 % to lower the misdiagnosis rate to the utmost extent, which resulted in a lower sensitivity. Nevertheless, additional studies on additional markers such as microRNAs should be performed to improve the sensitivity.

## Conclusions

In conclusion, using a multidisciplinary diagnostic team and based on results of histopathology and CSF’s cytology, we successfully determined the clinical diagnostic criteria of β-hCG and αFP in CSF and serum, which may greatly advance the early and formal diagnosis of ICGCTs.

## Abbreviations

αFP: alpha fetoprotein
β-hCG: β-subunit of human chorionic gonadotropin
BBB: blood-brain barrier
CSF: cerebrospinal fluid
FDG-PET: fluorodeoxyglucose-positron emission tomography
GCTs: germ cell tumors
ICT: intracranial tumors
ICGCTs: intracranial germ cell tumors
ITTs: intermediate trophoblastic tumors
LCH: langerhans cell histiocytosis
NGGCTs: non-geminomatous GCTs
ROC: receiver operating characteristic
STGCs: syncytial trophoblast giant cells
